# Enhanced diagnostic immunofluorescence using biopsies transported in saline

**DOI:** 10.1186/1471-5945-4-10

**Published:** 2004-08-27

**Authors:** Robert M Vodegel, Marcelus CJM de Jong, Hillegonda J Meijer, Marijn B Weytingh, Hendri H Pas, Marcel F Jonkman

**Affiliations:** 1 Centre for Blistering Diseases, Department of Dermatology, Groningen University Hospital, Groningen, the Netherlands

## Abstract

**Background:**

The demonstration of tissue-bound immunoreactants by direct immunofluorescence microscopy (DIF) is a valuable parameter in the diagnosis of various autoimmune and immunecomplex-mediated skin diseases. For preservation of tissue-bound immunoreactants, biopsies are usually fresh-frozen in liquid nitrogen or transported in Michel's fixative. But even optimally preserved tissue specimens are no guarantee for the correct diagnosis by DIF, especially when weak to moderate IgG fluorescence of the epidermal basement membrane zone is involved. In such cases false negative results are easily obtained due to the relatively high dermal "background" fluorescence produced by polyclonal anti-human IgG fluorescein conjugates.

**Methods:**

In the present study we have compared the use of normal saline (0.9% NaCl) with liquid nitrogen and Michel's fixative as transport medium for skin biopsies. From 25 patients with an autoimmune skin disease (pemphigus, pemphigoid, lupus erythematosus and vasculitis) four matched skin biopsies were obtained and transported in either saline for 24 and 48 hours, liquid nitrogen, or Michel's fixative for 48 hours.

**Results:**

Direct IF microscopy showed significant reduction of background fluorescence (p < 0.01) and relatively enhanced desired specific (IgG, IgA) staining in biopsies transported in saline. A conclusive or tentative IF diagnosis was reached in 92% after 24 h saline, 83% after 48 h saline, 68% after freezing in liquid nitrogen, and 62% after 48 h Michel's medium (n = 25).

**Conclusions:**

We conclude that transporting biopsies without freezing in normal saline for 24 hours is an adequate and attractive method for routine IF diagnosis in autoimmune and immune complex-mediated dermatoses. The superior results with saline incubation are explained by washing away of IgG background in dermis and epidermis.

## Background

The demonstration of tissue-bound immunoreactants by direct immunofluorescence microscopy (DIF) is a valuable parameter in the diagnosis of various autoimmune skin diseases[1]. Reliable diagnosis by DIF not only requires an experienced observer, but first of all proper skin (or mucosal) biopsies with well-preserved immunoreactants. For the latter purpose biopsies are usually snap-frozen in liquid nitrogen or, alternatively, placed in Michel's fixative that facilitates transport of biopsies from outside hospitals [2-6]. But even representative and optimally preserved tissue specimens are no guarantee for the correct diagnosis by DIF, especially when weak to moderate desired specific staining (DSS) of the epidermal basement membrane zone (BMZ) is involved [7]. In such cases, specific IgG fluorescence is easily masked by the relatively high dermal "background" fluorescence produced by polyclonal anti-human IgG fluorescein conjugates. The background fluorescence, consisting of both undesired specific staining (USS) and non-specific staining (NSS), largely determines the signal-to noise ratio [7]. This ratio in turn determines the detection threshold and thereby the diagnostic sensitivity of the DIF technique. A low ratio for IgG resulting from weak DSS and high USS plus NSS will undoubtedly yield false negative cases. So far, the signal- to noise ratio in diagnostic IF has received little attention.

The present study was initiated by the unexpected finding of significant increase of the signal-to noise ratio in a skin biopsy submitted for DIF and accidentally kept overnight in normal saline. The biopsy, obtained from a patient suspect of pemphigoid, showed substantial reduction of IgG background fluorescence and relatively bright specific IgG fluorescence along the BMZ. This finding encouraged us to compare diagnostic results of DIF in matched skin biopsies using standard snap-freezing, Michel's fixative and normal saline.

## Methods

### Patients

The 25 patients included in this study were selected on the basis of previously confirmed positive direct immunofluorescence (IF) in skin biopsies transported in liquid nitrogen. The final diagnosis was reached by clinical, routine laboratory, histological and direct IF findings. In case of bullous autoimmune diseases, serum samples were characterized by indirect IF on 1.0 M NaCl-split skin [8,9], immunoblotting [10], and ELISA (desmoglein 1 and 3) [11]. The patients had one of the following diagnoses: bullous pemphigoid (BP; n = 5); mucous membrane pemphigoid with skin involvement (MMP; 1); linear IgA dermatosis (LAD; 1), anti-epiligrin cicatricial pemphigoid (AECP; 1); epidermolysis bullosa acquisita (EBA; 1); dermatitis herpetiformis (DH; 1); pemphigus vulgaris (PV; 3); pemphigus foliaceus (PF; 3); subacute and systemic lupus erythematosus (LE; 5); and small vessel IgA vasculitis (4).

### Skin biopsies and processing

From each patient four skin specimens were obtained by punch biopsy (4 mm) using lidocaine as the local anaesthetic. The biopsies were taken from perilesional skin within an area of 2 cm^2 ^to minimize the risk of local variation of immunoreactants. The matched skin specimens from each patient were immediately placed in one of the following transport media: (a) liquid nitrogen, (b) Michel's fixative 48 hours with appropiate pH, (c) saline 24 hours and (d) saline 48 hours. We used 5 ml screw-capped polypropylene vials for transporting biopsies in Michel's fixative and saline.

### Freezing

Biopsies were placed in aluminum vials, snap-frozen in liquid nitrogen and stored at -80°C until further processing within two weeks for DIF (see below).

### Fixative

Michel's fixative and buffer solution were prepared monthly according to the original description.^1 ^Biopsy specimens were kept in 5 ml Michel's fixative for 48 hours (Mi48) at room temperature, followed by washing for 30 minutes in Michel's buffer solution. The specimens were then blotted on filter paper to remove excess moisture, and stored at -80°C until further processing.

### Saline

We used normal saline solution (0.9% NaCl in aqua dest.) without addition of calcium or magnesium. Skin specimens were kept in 5 ml saline solution for 24 hours (S24) and 48 hours (S48) at room temperature. Preliminary experiments with saline time of 6 hours did not result in improvement of the signal-to noise ratio. The specimens were then blotted on filter paper and stored at -80°C until further processing for DIF.

### Direct immunofluorescence microscopy

For comparative purposes, matched skin specimens of each patient were processed for direct IF microscopy at the same occasion. Cryosections of 4 μm thickness were mounted on polysine™ glass slides, air-dried for 30 min before a fan, and encircled with a hydrophobic emulsion (PAP-pen; DAKO; Glostrup). The sections were then stained for 30 min in a moist chamber at room temperature, using fluorescein (FITC)-labeled, Fc-specific goat F(ab')_2 _antibodies against human IgG, IgA and IgM (Protos Immunoresearch, Burlingame CA), and rabbit antibodies against human C3c and fibrinogen/fibrin (DAKO; Glostrup). Proper conjugate dilutions were made in phosphate-buffered saline (0.01 M PBS, pH 7.3) supplemented with bovine serum albumin 1%. After washing in 1000 ml PBS for 30 min, the sections were coverslipped under fresh PBS/glycerol (50% v/v). The slide preparations were kept at 4°C until microscopic examination within two days.

The sections were examined with a Leica DMRA microscope (Leica, Wetzlar, Germany) for selective incident light fluorescence using a xenon arc (XBO 75W) as light source and PL Apo ×40/0.80 dry objective. The fluorescent staining was graded as follows: - (negative), ± (doubtful), + (weak), ++ (moderate), +++ (bright). The desired specific staining (DSS) of the following target structures was scored^12;13^.: (a) epidermal in vivo ANA, (b) epidermal intercellular spaces, (c) basement membrane zone (dermo-epidermal junction), and (d) blood vessel walls. In addition, the background staining (USS plus NSS) of the upper dermis was scored. All sections were read blindly by the same experienced observer (MCJMdJ). The diagnosis made by direct IF was regarded as conclusive if the DSS score of the relevant target structure was at least weak but definite (Table). The diagnosis was regarded as tentative in case of weak staining that was not consistently distributed at the relevant target structure. A case was regarded as non-diagnostic if target structures showed negative to doubtful DSS scores.

Statistical analysis of IgG and IgA background fluorescence in matched biopsies was done by the McNemar test.

## Results

A total of 95 biopsies from 25 patients were examined; three biopsies were missing, and two biopsies proved unsuitable at cryosectioning. Cutting of 4 μm cryosections was easiest with biopsies transported in saline and hardest with biopsies transported in Michel's fixative.

**Figure 1:**
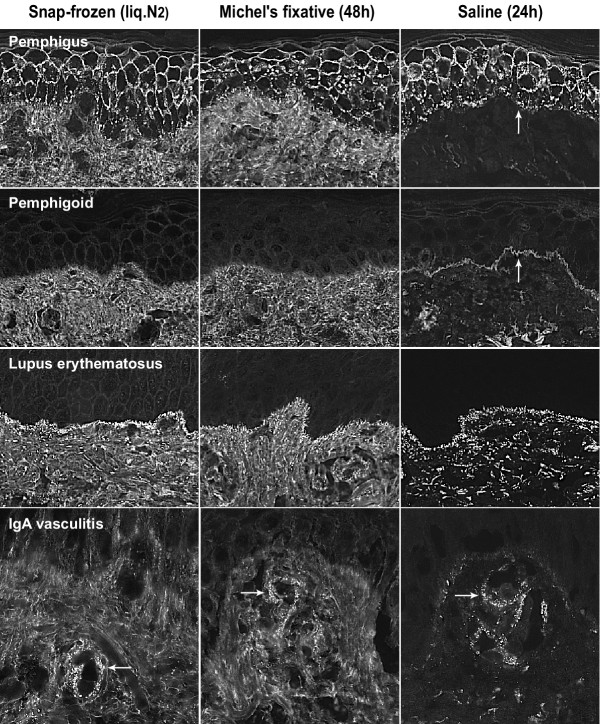
Comparison of direct immunofluorescence in cryosections of matched skin biopsies transported in liquid nitrogen, Michel's fixative or saline. Note the substantially reduced background fluorescence in saline-transported biopsies. Pemphigus foliaceus showing characteristic IgG fluorescence at the epidermal intercellular space. Additional granular IgG staining at the basement membrane zone (arrow) stands out most clearly in saline transported biopsy. (obj. ×20) Mucous membrane pemphigoid with skin involvement showing weak linear IgG fluorescence at the basement membrane zone that is only visible in the saline transported biopsy (arrow). (obj. ×20) Lupus erythematosus showing granular IgG fluorescence at the dermo-epidermal junction. Additional IgG staining of subepidermal vessel walls is best visible in the saline-transported biopsy. (obj. ×20) Vasculitis showing fine-granular IgA fluorescence in subepidermal capillary walls (arrows) which is most distinct in the saline-transported biopsy. (obj. ×40)

**Figure 2:**
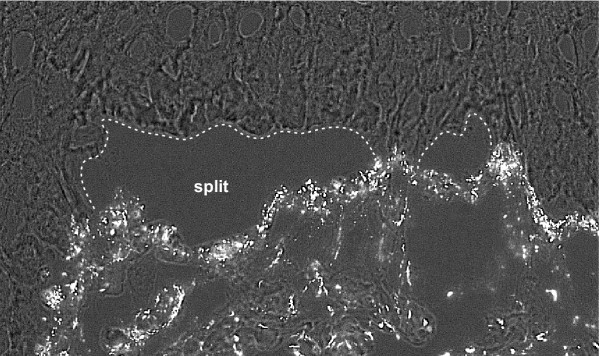
Direct immunofluorescence (IgG, combined with transmitted light) in saline transported skin specimen of lupus erythematosus. After 48 hours in saline there is subepidermal split formation, not present in fresh-frozen (N_2_) and fixed (Mi48) skin. Note the still obvious granular IgG fluorescence at the dermal side of the split. (obj. ×40)

Biopsies kept in saline for 24 and 48 hours showed statistically significant reduction of background fluorescence in the dermis, especially with IgG, as compared with fresh-frozen and fixed biopsies (p < 0.01). After 24 hours, this resulted in enhanced signal-to noise ratios and accordingly more easy detection of immunoreactants at target structures, in particular the epidermal basement membrane zone (BMZ) and subepidermal blood vessel walls (Fig. 1). In comparison, fresh-frozen and fixed biopsies showed a relatively poor signal- to noise ratio for IgG (and IgA). In these biopsies, weak (+) specific fluorescence of IgG at the BMZ or IgA in vessel walls was found to be masked easily by relatively high background staining. Biopsies kept for 48 hours in saline showed a variable degree of diminution of specific staining, and tended to become negative in case of weak (+) specific fluorescence. The IgG fluorescence of epidermal in vivo ANA, present in fresh-frozen and fixed biopsies of two cases with (S)LE, became negative in saline biopsies (both 24 h and 48 h). In general, biopsies with moderate to bright (++/+++) specific fluorescence remained positive in all transport media. The reduced background fluorescence in saline biopsies occasionally revealed doubtful to weak focal IgG fluorescence of the BMZ that was regarded as non-relevant. Furthermore, we observed dermo-epidermal split formation in saline biopsies, not present in matched fresh-frozen and fixed biopsies. The extent of split formation varied among saline biopsies and increased with time (48 h > 24 h). In cases with pemphigoid, IgG was found predominantly at the epidermal side of splits, in contrast with e.g. SLE where IgG was found at the dermal side (Fig. 2). In case an artificial split is induced because of saline incubation a BMZ signal stand more out because of the dark background of the blister cavity (Fig. 2). The overall morphology in saline biopsies was quite fair and largely sufficient for the purpose of diagnostic IF microscopy.

The diagnostic results of direct IF in matched biopsies are summarized in Table I. By interpreting these data it should be realized that the minority of cases (20%) showed bright (+++) specific fluorescence in standard frozen biopsies, whereas the majority (52%) showed only weak to moderate (+/++) specific fluorescence of the relevant target structure(s). Two originally positive cases, one pemphigus and one IgA vasculitis became doubtful or negative (non-diagnostic) in all transport media. The highest rate of conclusive cases by direct IF was obtained in biopsies kept in saline for 24 hours (S24, 84%), and the lowest in fixed biopsies (Mi48, 50%). The case of mucous membrane pemphigoid with weak IgG (+) and IgA (+) fluorescence at the BMZ was obvious in saline biopsies (S24, S48) but non-diagnostic in matched fresh-frozen and fixed biopsies. The highest non-diagnostic percentage was obtained in fixed biopsies (32%) and the lowest in S24 saline biopsies (8%). In one case, classified as misdiagnosed, the fresh-frozen biopsy led to the diagnosis of LAD as IgA (+) and C3c (+) were the only immunoreactants observed, whereas the matched fixed and saline biopsies showed additional linear IgG (+/++) fluorescence at the BMZ (suggestive of mixed IgG/IgA pemphigoid). After correction for the missing biopsies, the results were only statistically significant comparing Mi and S24 (p < 0.05). Other comparisons (N2 versus Mi, S24 and S48 or Mi versus S48 or S24 versus S48) were not significant.

**Table 1:** Results of direct immunofluorescence (DIF) of matched skin biopsies transported in different media.

		**N2**	**Mi48**	**S24**	**S48**
	
**Diagnosis by DIF**	DSS	n = 25	n = 22^§^	n = 25	n = 23^§^
a. conclusive	+/+++	14 (56%)	11 (50%)	21 (84%)	16 (70%)
b. tentative	**±/+**	5 (20%)	4 (18%)	2 (8%)	3 (13%)
c. non-diagnostic	**-/±**	5 (20%)	7 (32%)	2 (8%)	4 (17%)
d. mis-diagnosed		1 (4%)	-	-	-

N_2_, snap-frozen specimens in liquid nitrogen; Mi48, specimens in Michel's fixative for 48 hours; S24 and S48, specimens in saline for 24 and 48 hours respectively; DSS, desired specific staining; n, number of cases

^§ ^Two S48 and three Mi48 matched biopsies were either lacking or considered unsuitable.

## Discussion

Perilesional skin biopsies kept in saline for 24 hours yielded a higher diagnostic rate in direct IF than fresh-frozen biopsies in liquid nitrogen or biopsies kept in Michel's fixative.

Several authors have described the positive effect of saline in increasing the sensitivity of IF analysis. Judd and Lever showed that skin biopsies stored for 24 hours in 0.15 M phosphate buffered saline prior to freezing gave a very high incidence of positive readings in direct IF [14]. Similarly, the use of 1.0 M NaCl split skin as a diagnostic tool for direct and indirect IF has been reported to increase the sensitivity of these methods [9,15-17]. It has been suggested that the increase of IF sensitivity by saline incubation is due to improved exposure of epitopes and/or by a decrease of background staining [16].

Our data suggest that the improved DIF sensitivity in saline biopsies is primarily due to decreased background staining resulting in better image contrast (signal-to noise ratio). We know that our visual perception is sensitive for light contrast and not for intensity [18]. The staining intensity perceived of a given specific fluorescent signal is strongly correlated with the intensity of the background (compare looking at the stars in daylight and at night; the intensity is the same, but seems multiplied at night). In this respect, IgG (and IgA) fluorescence in skin tissue is hampered by relatively strong background fluorescence in the dermis that may mask weak specific fluorescence at the BMZ and in vascular walls. Saline appears to reduce this background staining, resulting in relative increase of desired specific staining and thereby enhanced diagnostic sensitivity.

A disadvantage of saline is the limited time of transport (24 h) for consistently reliable results. If biopsies are kept longer than 24 hours in saline, decreased fluorescence of tissue-bound immunoreactants may be encountered, although we have observed bright specific fluorescence of the BMZ in biopsies kept in saline for at least 5 days. Michel's fixative seems to have a similar limitation: Skeete and Black found that biopsies stored in this fixative should be received within 1 day of biopsy for consistently reliable results [4].

The second disadvantage of saline is the loss of at least some epidermal *in vivo *ANAs, possibly due to extraction or degradation of nuclear antigens. Another disadvantage of saline might be, from a histopathological point of view, morphological disturbances such as hydropic degeneration [19], and splitting at the dermo-epidermal junction, not found with Michel's fixative [6]. That is why saline is not suitable for antigen mapping in the diagnosis of genetic diseases [20]. Neither are biopsies kept in saline suited for immuno-electron microscopy [5,6,20]. However, for the diagnosis of autoimmune and immune complex-mediated diseases by DIF, it is not optimal morphology that counts, but the low detection threshold of immunoreactants. In this regard, (artificial) dermo-epidermal split-formation may add value to the method, rather than being a problem, by darkening of the juxtaposed background and mapping of the linear epidermal BMZ deposition [21].

Besides the diagnostic benefit, the preference would also go to the use of saline because it is a ready available, inexpensive, and convenient transport medium, and it certainly improves cutting properties of skin biopsies compared to biopsies fixed in Michels' medium. Saline can be used as standard medium at room temperature for express postal delivery of IF biopsies to the laboratory, if delivery is guaranteed within 24 hours. Transport of biopsies by express postal delivery or in-house airtube post is much more cost-effective than courier delivery necessary for biopsies transported in liquid nitrogen. If it is expected to tide over a longer period we advise to store the biopsy in saline for 24 hours and then to place it in Michel's fixative for further transport.

Practically, place the biopsy in a screw-capped 5 ml polypropylene tube filled to the top with saline. The saline does not have to be sterile. If the specimen arrives in the laboratory the same day after biopsy, we advise to keep it overnight in saline at room temperature, followed by snap-freezing and (optional) storage at -80°C the next day.

## Conclusions

The use of normal saline offers an attractive alternative to liquid nitrogen and Michel's fixative for diagnostic IF in autoimmune and immune complex-mediated dermatoses, but is only consistently reliable if the specimens are received within 24 hours after biopsy.

## Competing interests

None declared.

## Abbreviations

BMZ: basement membrane zone

BP : bullous pemphigoid

DH: dermatitis herpetiformis

DIF: direct immunofluorescence microscopy

DSS: desired specific staining

EBA: epidermolysis bullosa acquisita

IF: immunofluorescence microscopy

LAD: linear IgA dermatosis

LE : lupus erythematosus

MMP: mucous membrane pemphigoid

NSS: non-specific staining

PF : pemphigus foliaceus

PV : pemphigus vulgaris

USS: undesired specific staining

## Authors' contributions

RV carried out the studies, participated in the design and data analysis and drafted the manuscript. MdJ participated in patient selection and carried out the immunofluorescence microscopy and imaging. HM performed the immunofluorescence technical work up. MW participated in material harvesting. HP participated in patient diagnosis. MJ conceived the study, and participated in its design and coordination. All authors read and approved the final manuscript.

## Pre-publication history

The pre-publication history for this paper can be accessed here:


